# Treatment of a Patient with Acute Promyelocytic Leukemia with Multiple Isolated Relapses in the Central Nervous System: A Case Report and Mini-Review of the Literature

**DOI:** 10.1155/2024/5593775

**Published:** 2024-05-03

**Authors:** Qixin Sun, Wenyi Chen, Ahui Wang, Zili Yang, Guiping Chen, Zhigang Zhu

**Affiliations:** ^1^Departments of Geriatric Hematology and Oncology, Guangzhou First People's Hospital, Guangzhou, Guangdong 510180, China; ^2^Departments of Medical Records, Guangzhou First People's Hospital, Guangzhou, Guangdong 510180, China; ^3^Departments of Clinical Laboratory, Guangzhou First People's Hospital, Guangzhou, Guangdong 510180, China; ^4^Departments of Geriatric Critical Care Medicine, Guangzhou First People's Hospital, Guangzhou, Guangdong 510180, China

## Abstract

The efficacy of therapeutics for acute promyelocytic leukemia (APL) has exhibited an increase in recent years. Only a few patients experience relapse, including extramedullary relapse, and in patients with extramedullary relapse, the central nervous system (CNS) is the most common site. To date, there is no expert consensus or clinical guidelines available for CNS relapse, at least to the best of our knowledge. The optimal therapeutic strategy and management options for these patients remain unclear. The present study reports the treatment of a patient with APL with multiple isolated relapses in the CNS. In addition, through a mini-review of the literature, the present study provides a summary of various reports of this disease and discusses possible treatment options for these patients.

## 1. Introduction

The overall efficacy of therapeutics for patients with acute promyelocytic leukemia (APL) has significantly improved over the past two decades [1, 2]. Only a small number of patients experience relapse, and the number of patients with multiple isolated central relapses is even smaller [3]. To the best of our knowledge, there are currently no available clinical guidelines to follow in the case of relapse of this disease. The present study reports the treatment of a patient with APL with multiple isolated relapses in the central nervous system (CNS) and also describes the disease characteristics of this patient. In addition, the present study provides a summary of various reports of this disease and discusses possible treatment options for these patients, by performing a mini-review of the literature.

## 2. Case Report

The present study reports the case of a 47-year-old female patient with multiple petechiae and ecchymosis for two weeks in December, 2016. Following routine peripheral blood tests, the number of platelets was found to be 18 × 10^9^/l and ∼50% immature cells could be found in the blood. Considering the possibility of acute leukemia, she was admitted to the Guangzhou First People's Hospital for further treatment. A series of bone marrow tests were performed according to the leukemia MICM classification (WHO 2016) [4].

The bone marrow (BM) was hypercellular with 86% promyelocytes. The karyotype was normal; however, fluorescence *in situ* hybridization (FISH) analysis revealed a *PML/Rara* fusion gene ratio of 91%; immunophenotyping analysis revealed positive results for the CD117, CD34, CD13, and CD33 antigens and negative results for the HLA-DR, CD3, CD4, CD5, CD8, CD10, CD19, CD20, CD11b, CD64, and CD15 antigens. The results of quantitative PCR (qPCR) revealed *PML/Rara: ABL* = 47.98% (S type); next-generation sequencing (NGS) analysis revealed *FLT3-ITD* and *ECT2L* gene aberrations. Thus, in consideration of these results as whole, she was diagnosed with APL.

The patient received all-trans retinoic acid (ATRA) at 45 mg/m^2^ daily as the basic induction protocol, and idarubicin (IDA) at 8 mg/m^2^ was administered every other day when the white blood cell count was <10 × 10^9^/l, with a total of three times. Disseminated intravascular coagulation and differentiation syndrome were not observed during the whole induction period. On day 33 of induction, bone marrow morphology indicated complete remission (CR). Subsequently, the patient's cerebrospinal fluid (CSF) was examined using BD flow cytometry (FCM) and a prophylactic intrathecal injection (dexamethasone, 5 mg; cytarabine, 50 mg) was routinely administered. She was discharged after the CSF test (FCM and morphology) yielded negative results.

The whole consolidation therapy period was divided into four cycles. Each cycle included the following: (i) ATRA at 45 mg/m^2^/day, administered orally, from day 1 to day 14; (ii) arsenic trioxide (ATO) 10 mg/day administered intravenously, from day 15 to day 28; and (iii) IDA at 8 mg/m^2^/day administered intravenously, from day 29 to day 31. CSF testing was performed and a prophylactic intrathecal injection was routinely administered at the end of each cycle.

Bone marrow minimal residual disease (MRD) was performed prior to the initiation of each cycle. *PML/Rara* fusion gene molecular negative was observed at the beginning of the second consolidation treatment, and the results continued to be negative in each subsequence test, including at the time of the relapse in the CNS.

Maintenance therapy included an all-oral drug regimen and was planned to continue for 2 years in eight cycles. Each cycle would last for 3 months and would include the following: first month—ATRA at 45 mg/m^2^ from day 1 to day 14; second month—oral arsenic (Realgar-Indigo naturalis formula), 60 mg/kg daily in divided doses from day 1 to day 28; third month—6-mercaptopurine tablets (100 mg/day) from day 1 to day 14. At the end of each treatment cycle, bone marrow MRD was performed using qPCR. The patient responded well to this treatment plan until the fifth cycle of maintenance. She then exhibited transient eye pain, which improved after being examined and treated as glaucoma by an ophthalmologist.

The first definite CNS relapse occurred at the sixth cycle of maintenance, at ∼24 months from the time of the diagnosis of APL. The typical symptoms were severe headaches and eye pain while there was no abnormality in the brain by MR scan. A large number of immature cells were detected in the CSF using FCM. However, at the same time, bone marrow *PML/Rara* fusion gene tests using qPCR continued to yield negative results.

**Figure 1:**
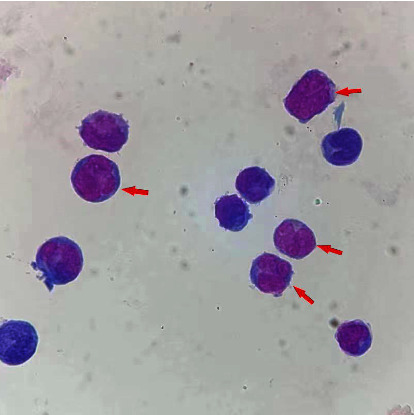
Cytomorphology of CSF during the second relapse, red arrow (immature cell) (10 × 100).

Subsequent salvage therapy was performed, which included three drugs combined in an intrathecal injection (methotrexate at 10 mg, cytarabine at 50 mg, and dexamethasone at 5 mg) administered twice a week and ATO at 10 mg/day administered via intravenous infusion. The patient's symptoms rapidly dissipated, and the intrathecal injection was terminated when the CSF FCM-MRD tests yielded negative results for five consecutive times. The total number of intrathecal injections and the duration of ATO treatment administered for this relapse were 14 times and 30 days, respectively. Subsequently, in order to consolidate the remission status, the patient was administered ATO at 10 mg/day for 2 consecutive weeks once a month, together with a CSF test and prophylactic intrathecal injection. However, 11 months later, the patient suffered the second CNS relapse (Figure 1), which was identified using a routine CSF cytomorphology test, as the patient was asymptomatic. At this time, brain scans still showed no abnormalities and the bone marrow *PML/Rara* fusion gene test results continued to be negative. Therefore, HSCT was still not considered in our salvage scheme.

**Figure 2:**
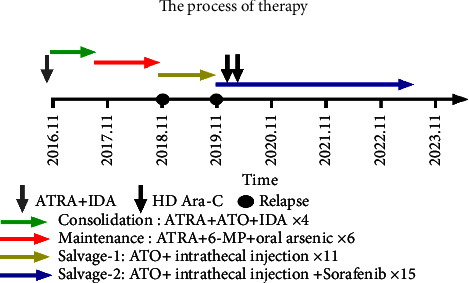
The process of therapy.

In fact, the new scheme was similar to that of the first protocol, with the only difference being that sorafenib at 0.4–0.8 g/day was administered orally, according to the changes in routine blood counts. In addition, in order to prevent further relapse, twice additional high-dose Ara-C (21 g) chemotherapy was also administered after the CNS leukemia remission period. To date, the frequency of ATO infusion and prophylactic intrathecal injection has been extended from quarterly to semiannually, although sorafenib treatment was never terminated. The latest results have indicated that the patient continues to be still in CR, as per bone marrow and CSF tests. It has been 49 months since the second remission (Figure 2).

## 3. Discussion

Currently, APL exhibits optimal therapeutic response of all types of leukemia. With the use of ATRA and arsenic, the molecular CR and overall survival of patients are markedly increased [5]. A recent study demonstrated that the 13-year overall survival rate of patients was >90% [1]. In addition, the early use of arsenic in the induction, consolidation, and maintenance stages, particularly with the emergence of oral arsenic [6], has not only improved the therapeutic efficacy [7, 8] but also facilitated patients and improved their quality of life. Although some opinions may differ, a novel all-outpatient treatment mode has often been reported in recent years [6, 9, 10], which is particularly suitable for patients who are classified as low-middle risk.

NGS testing has been widely used in the diagnosis of acute myeloid leukemia, and the role of certain mutated genes such as *FLT3-ITD* in risk stratification and therapeutic guidance in acute myeloid leukemia has been wildly recognized [11–13]. However, for patients with APL, the impact of gene mutations remains unclear [14]. This may be due to the fact that almost all patients with APL exhibit a good response to ATRA and arsenic treatment, even those having some gene mutations at the time of the first diagnosis [15]. Thus, the majority of research has reported high-risk mutant genes identified in acute myeloid leukemia, such as *FLT3-ITD*; to date, there are no clinical data on the role of gene mutations in the treatment of APL [16, 17].

However, with the increasing number of patients exhibiting disease-free-survival, some patients with late relapse have been observed in clinical practice [18], particularly those with CNS relapse; the majority of these patients have *FLT3-ITD* gene mutations or other high-risk factors, including cerebral hemorrhage, hyperleukocytosis syndrome in the induction period, a high expression of adhesion molecules (CD56) induced by ATRA treatment, and the bcr3 subtype of the *PML/Rara* fusion gene [19–21]. However, compared with the number of patients with APL exhibiting CR and long-term survival, the number of patients experiencing relapse is almost negligible. To date, at least to the best of our knowledge, no relevant clinical trials have been launched including such patients [22].

As is known, CNS leukemia is a common complication associated with the treatment of acute leukemia, particularly in acute lymphoblastic leukemia (ALL). The therapeutic options include intrathecal chemotherapy (by lumbar puncture or Ommaya reservoirs), high-dose Ara-C or MTX chemotherapy, total brain and spinal cord irradiation, and even CAR T-cells for B-cell ALL [22]. These treatments may be used for patients with APL with CNS leukemia. However, the majority of clinical data demonstrate that it is difficult to obtain long-term survival without subsequent radical treatment, such as hematopoietic stem cell transplantation [23, 24].

APL cells are particularly sensitive to arsenic and some relapses are limited to the CNS. Thus, the identification and development of strategies which will ensure that higher levels of arsenic enter the CNS, and which can promote and induce higher levels of APL cell apoptosis, have become a therapeutic direction for APL CNS leukemia. An unusual strategy would involve the direct injection of arsenic into the CSF via a lumbar puncture needle; however, this would pose a number of ethical and legal issues. To date, such a strategy has not been reported in clinical practice.

In fact, previous studies have reported that arsenic can penetrate the blood-brain-barrier on its own [25, 26], although at a very low concentration [20]; the ratio of CSF/plasma arsenic concentration has been shown to be 10–20%, with substantial differences observed among individuals [27]. Some researchers have reported high concentrations of arsenic and differentiation-syndrome-like phenomena in the CSF of patients, particularly in those with a history of irradiation treatment [28, 29]. However, it is considered that this is more likely the result of the blood-brain barrier being destroyed by irradiation. Furthermore, there are reports of carotid artery catheterization injection or the rapid peripheral intravenous infusion of mannitol; these can also overcome the blood-brain barrier and increase arsenic concentration in the CSF.

The strategy of the peripheral intravenous rapid infusion of mannitol has been previously reported in the literature; the key steps involved a 20% mannitol intravenous bolus (125 ml, 12–30 ml/min) and bridge mixed mannitol (250 ml) and arsenic (5 mg) via rapid drip (6 ml/min), which can noninvasively and transiently open the blood-brain barrier [30, 31]. However, it is considered that this “off-label” usage should be performed in patients with a better cardiac function; at the same time, there may be a potential risk of epileptic seizure induced by the excessive penetration of arsenic into the CNS.

**Figure 3:**
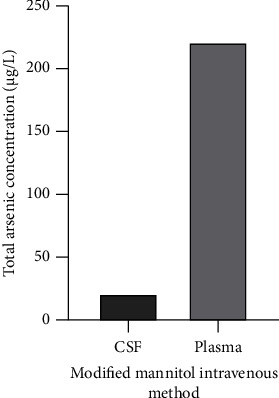
Total arsenic concentration in plasma and CSF during the arsenic treatment stable period by modified mannitol intravenous method at the second relapse.

This protocol was not followed completely for the patient in the present study. During the daily infusion of arsenic, only another intravenous infusion channel was made for rapid mannitol infusion for one to two times. Based on the authors' observations, the arsenic concentration in the CSF did not exhibit a notable increase with this modified method. The ratio of the CSF/plasma arsenic concentration was ∼1 in 10 (21.4 vs. 221.3 *μ*g/l) (Figure 3). Of note, the total CR2 period with this treatment was only 11 months, which also indicated that this modified method could not increase the intracranial diffusion of arsenic and that it was difficult to treat leukemia cells in the CNS.

To date, there are no guidelines available for the treatment of patients with this disease condition. According to the general consensus, under the base of intrathecal chemotherapy and arsenic infusion, combined treatment with sorafenib was also performed, which targets *FLT3-ITD*. APL is a rare subtype of acute myeloid leukemia. Although there are no studies available in the literature to date supporting the use of sorafenib to target *FLT3-ITD* in APL, the use of sorafenib to target *FLT3-ITD* in patients with non-APL acute myeloid leukemia before and after transplantation has become a general consensus [32].

In addition, an increasing number of small-molecule targeted drugs have been shown to lead to an improved therapeutic response in CNS malignant disease. These include the notable therapeutic effect of BTK inhibitors for CNS lymphoma with *MYD88* mutation [33]. In addition, it was previously demonstrated that the incidence of CNS leukemia was significantly reduced in Ph + ALL by tyrosine kinase inhibitor [34]. Other studies have demonstrated that patients with APL with CNS leukemia can be successfully treated with Bcl-2 inhibitors (venetoclax) [35, 36]. Recently, gilteritinib-based regimens have also been reported to be effective in the treatment of APL with FLT3-ITD mutation extramedullary relapse [37]. All these findings support the use of small-molecule targeted agents for APL with CNS leukemia.

To date, the latest CR3 period of the patient in the present study is > 49 months; the MRD tests of CSF (morphology and immunology) have consistently yielded negative results. Compared with the 11 months of the CR2 period, the remission period was significantly prolonged with the addition of sorafenib. As far as we know, this is the first report that confirmed sorafenib-containing regimen was effective in APL CNS leukemia with FLT3-ITD mutation and it is worth more verification in the future.

## Data Availability

The datasets used and/or analyzed during the current study are available from the corresponding author on reasonable request.
